# Segmentation and classification of two-channel C. elegans nucleus-labeled fluorescence images

**DOI:** 10.1186/s12859-017-1817-3

**Published:** 2017-09-15

**Authors:** Mengdi Zhao, Jie An, Haiwen Li, Jiazhi Zhang, Shang-Tong Li, Xue-Mei Li, Meng-Qiu Dong, Heng Mao, Louis Tao

**Affiliations:** 1 Center for Quantitative Biology, Academy for Advanced Interdisciplinary Studies, Peking University, Yiheyuan Road, Beijing, 100871 China; 2 LMAM, School of Mathematical Sciences, Peking University, Yiheyuan Road, Beijing, 100871 China; 3 Center for Bioinformatics, National Laboratory of Protein Engineering and Plant Genetic Engineering, School of Life Sciences, Peking University, Yiheyuan Road, Beijing, 100871 China; 4 National Institute of Biological Sciences, Beijing, Kexueyuan Road, Beijing, 102206 China

**Keywords:** *C. elegans*, Nucleus, Aging, Two-channel fluorescence image, Morphology, Segmentation, Classification

## Abstract

**Background:**

Aging is characterized by a gradual breakdown of cellular structures. Nuclear abnormality is a hallmark of progeria in human. Analysis of age-dependent nuclear morphological changes in *Caenorhabditis elegans* is of great value to aging research, and this calls for an automatic image processing method that is suitable for both normal and abnormal structures.

**Results:**

Our image processing method consists of nuclear segmentation, feature extraction and classification. First, taking up the challenges of defining individual nuclei with fuzzy boundaries or in a clump, we developed an accurate nuclear segmentation method using fused two-channel images with seed-based cluster splitting and k-means algorithm, and achieved a high precision against the manual segmentation results. Next, we extracted three groups of nuclear features, among which five features were selected by minimum Redundancy Maximum Relevance (mRMR) for classifiers. After comparing the classification performances of several popular techniques, we identified that Random Forest, which achieved a mean class accuracy (MCA) of 98.69%, was the best classifier for our data set. Lastly, we demonstrated the method with two quantitative analyses of *C. elegans* nuclei, which led to the discovery of two possible longevity indicators.

**Conclusions:**

We produced an automatic image processing method for two-channel *C. elegans* nucleus-labeled fluorescence images. It frees biologists from segmenting and classifying the nuclei manually.

**Electronic supplementary material:**

The online version of this article (doi:10.1186/s12859-017-1817-3) contains supplementary material, which is available to authorized users.

## Background

The nucleus is vital for many cellular functions and is a prominent focal point for regulating aging [1–3]. *Caenorhabditis elegans* (*C. elegans*) is an important model organism for studying aging because of its small size, transparent body, well-characterized cell types and lineages. Several important studies have found age-related morphological alterations in *C. elegans* nucleus, such as changes of nuclear shape and the loss of peripheral heterochromatin [4]. It is reported that these alterations are highly related to lamin and chromatin. Therefore, biologists usually label them with fluorescence proteins and use the fluorescence images to study aging [5–8].

To assess characteristics of nuclear morphology during the aging process, biologists usually manually identify the nuclei from images, subjectively estimate the type of the nuclei and evaluate the nuclear morphology according to experience. This process lacks consistent standards and high efficiency. Thus, an effective and automatic processing method for *C. elegans* fluorescence images is needed for nuclear morphological analysis.

There is a rapid development of imaging informatics, producing some advanced segmentation and classification methods [9–16]. We have tried these methods and found that many of them do not work properly on our images because of the complexity of our images. In our images, many nuclei are highly textured, leading to low intensity continuity and messy gradient directions. Furthermore, our images have a wide range of nuclear sizes, covering both small nuclei (neuronal nuclei) and large nuclei (intestinal nuclei). The high background noise and large variation of image quality also affect the segmentation results. Thus, the existing methods are not suitable for our images. More details of these method’s limitations and discussions can be found in Additional file 1. In addition, few image processing studies and quantification researches focus on *C. elegans* nucleus-labeled fluorescence images, not only because of the gap between biology and image processing field, but also the image processing challenges.

Age-related changes of nuclear architecture of *C. elegans* pose a challenge to image analysis. Extensive deterioration of the nuclear morphology has been observed in worms of advanced age, including a systemic loss of DAPI-stained intestinal nuclei, which could result from loss of nuclei, loss of nuclear DNA, or reduced affinity of old DNA for DAPI for unknown reasons [17]. Identifying intestinal nuclei by green fluorescent protein (GFP) labeling also becomes ineffective in old worms due to an increase of background fluorescence [18]. In addition, images of old *C. elegans* nuclei are intrinsically fuzzier and misshapen, because old nuclei lose their round shape and their proper distribution of nuclear components [19]. As such, despite the rapid development of imaging informatics, processing methods that can handle fluorescence images of both young and old *C. elegans* nuclei are currently unavailable.

In this paper, we present an integrated image processing method on two-channel nuclear-labeled fluorescence image. First, a segmentation method based on two-channel images fusion is proposed to separate the nuclei from the background. Second, a set of geometric, intensity and texture features are extracted to describe nuclear morphological properties. Five features are selected by mRMR as the most important features for classification. Next, several classification algorithms are employed and compared. Finally, two examples of quantitative feature analysis are shown.

## Methods

**Fig. 1:**
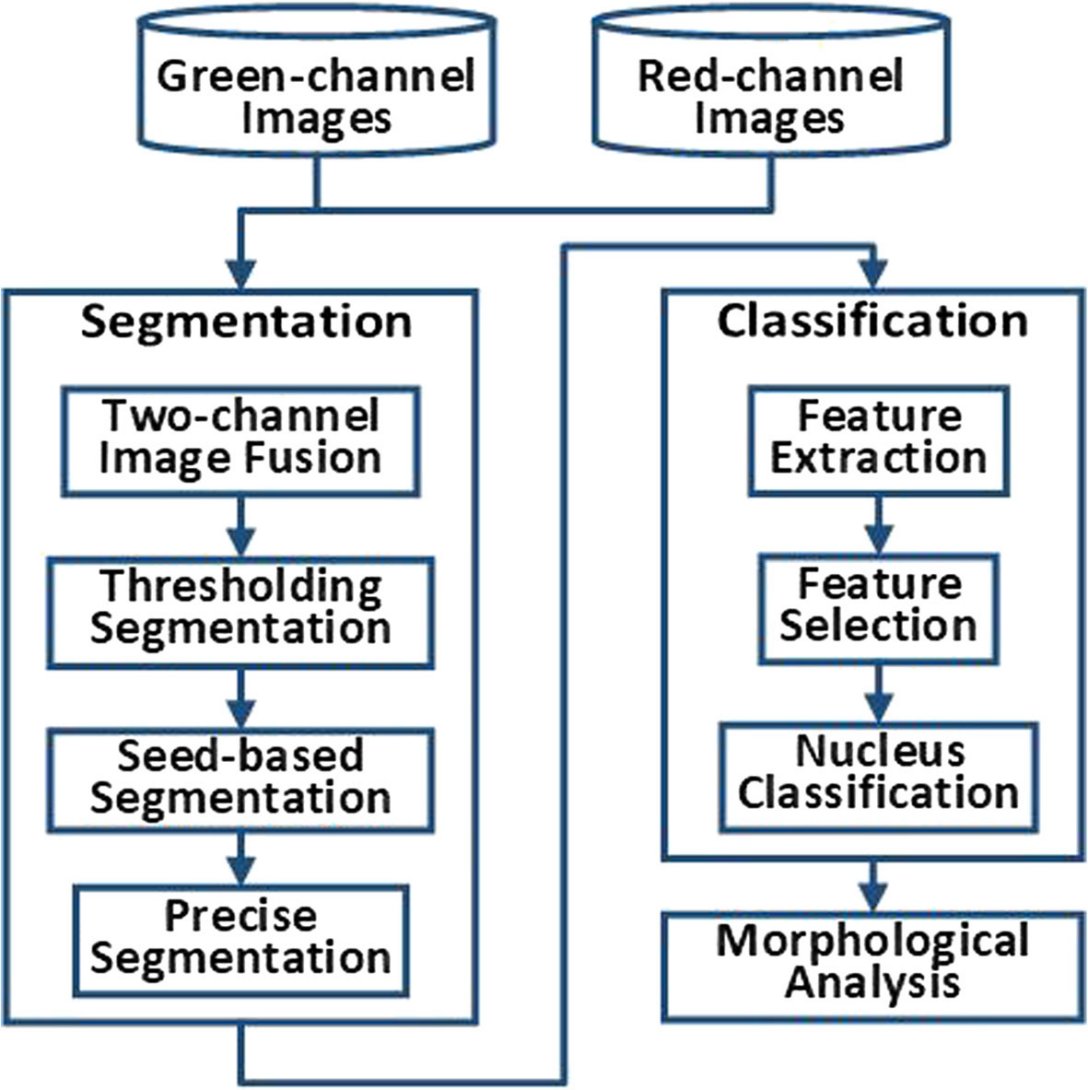
Flowchart of the image processing approach. Green-channel images and red-channel images are input into nucleus segmentation. Two-channel images are fused together for further thresholding segmentation, seed-based segmentation and precise segmentation. Next, several features are extracted from the segmented nucleus and are filtered by feature selection. Then, the selected features are applied for classification. Finally, the classified images are quantified for morphological analysis

In this section, the acquisition and processing method of *C. elegans* nucleus-labeled fluorescence images are presented in detail. Figure 1 shows the flowchart of the method.

### *C. elegans* strains

The two *C. elegans* strains used in this study were MQD1658 and MQD1798. They both express LMN-1::GFP, which labels nuclear lamina with green fluorescence, and HIS-72::mCherry, which labels histone with red fluorescence, either in the wild type background (MQD1658) or in the long-lived *daf-2(e1370)* background (MQD1798). MQD1658 was constructed by crossing LW697 *ccIs4810 [lmn-1p::lmn-1::gfp::lmn-1 3’utr + (pMH86) dpy-20(+)]* with XIL97 *thu7[his-72::mCherry]* and selecting for double homozygous offspring. MQD1798 was obtained by crossing MQD1658 with CF1041 *daf-2(e1370)* and selecting for triple homozygous offspring.

Genotype of MQD1658: *thu7 [his-72::mCherry]*; *ccIs4810 [lmn-1p::lmn-1::gfp::lmn-1 3’utr + (pMH86) dpy-20(+)]*.

Genotype of MQD1798: *daf-2(e1370)*; *thu7 [his-72::mCherry]*; *ccIs4810 [lmn-1p::lmn-1::gfp::lmn-1 3’utr + (pMH86) dpy-20(+)]*.

### Image acquisition

**Fig. 2:**
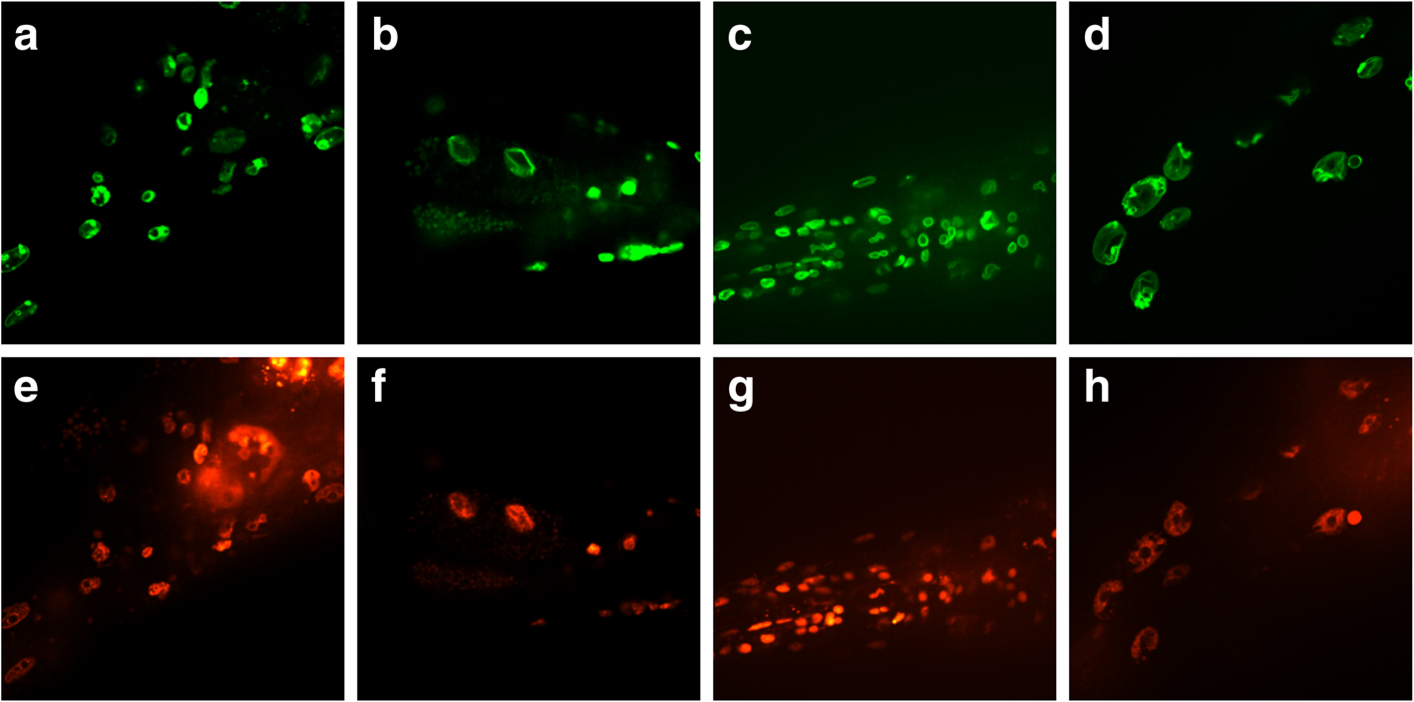
Fluorescence images acquired using 488-, 561-nm excitation. **a**-**d** are the green-channel images, indicating nucleus membrane. **e**-**h** are the corresponding red-channel images, indicating chromosome

The image acquisition method is essentially the same as described previously [20]. Worms were cultured under standard conditions, i.e. at 20°C on NGM plates seeded with OP50 *E. coli*. Worms were anesthetized with 1 mM levamisole on an agarose pad before being imaged using a spinning-disk confocal microscope (UltraVIEW VOX; PerkinElmer) equipped with a 63 ×, 1.4 numerical aperture (NA) oil-immersion objective. LMN-1::GFP and HIS-72::mCherry signals were excited at 488 nm and 561 nm, and collected at 500-550 nm and n nm, respectively. The exposure time and laser power were varied to balance the fluorescence intensity among samples. All images were transformed into TIF format and cropped into 1000 × 1000 array. Figure 2 shows the examples of the images.

**Fig. 3:**
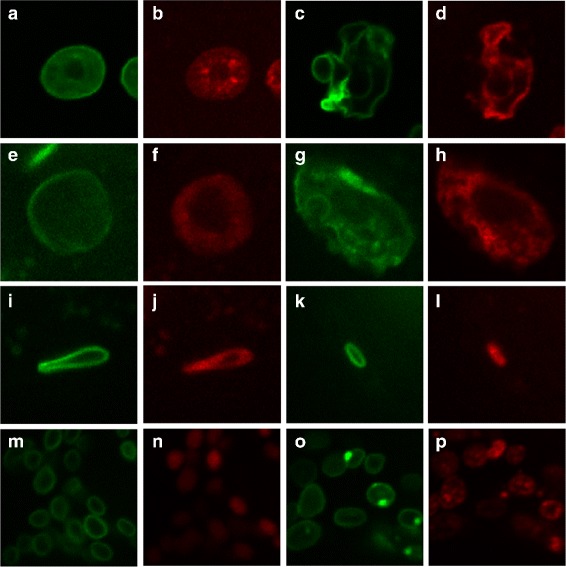
Four types of *C. elegans* nucleus in day 1 and day 16. Images in the same row are the same nuclear types: (**a**-**d**) hypodermal nuclei, (**e**-**h**) intestinal nuclei, (**i**-**l**) muscle nuclei and (**m**-**p**) neuronal nuclei. Images in first two columns are the green-channel and red-channel images captured in day 1. Images in the third and fourth columns are captured in day 16

Our image set contains 1364 groups of images from two *C. elegans* strains with different ages in days 1, 4, 6, 10, 12, 14, 16. Table 1 describes the amount of image groups of two strains in each day. Each group includes one green-channel image and one red-channel image. The green channel indicates nuclear membrane and the red channel chromosome. In this work, we restrict our attention to four types of nuclei: hypodermal, intestinal, muscle and neuronal nuclei. Figure 3 shows the examples of four types of nuclei in day 1 and day 16.

**Table 1:** The amount of images of different strains and ages

Strain	Day1	Day4	Day6	Day10	Day12	Day14	Day16
wild type	122	116	102	72	119	105	97
*daf-2(e1370)*	80	114	86	61	119	79	92

### Nuclear segmentation

This section describes how we segment nuclei from the background. From the examples in Fig. 2, we can see that there is much noise from the fluorescence of neighboring nuclei and some nuclei cluster closely together. Thus the fuzzy boundary and clustered nuclei are the two main challenges in nuclear segmentation. Considering these challenges, we propose a method to effectively separate the nucleus from the noisy background and adjacent nuclei. The procedure consists of four steps: two-channel image fusion, thresholding segmentation, seed-based segmentation and precise segmentation.

#### Two-channel image fusion

In our imaging data, green-channel images are more reliable than red-channel images, because the former are clearer and have higher signal-to-noise ratio (more details can be found in Additional file 1). Even though the green-channel images are reliable, they have low intensity and fuzzy boundaries. Thus, we fuse green-channel and red-channel images to enhance the contrast of nuclei.

**Fig. 4:**
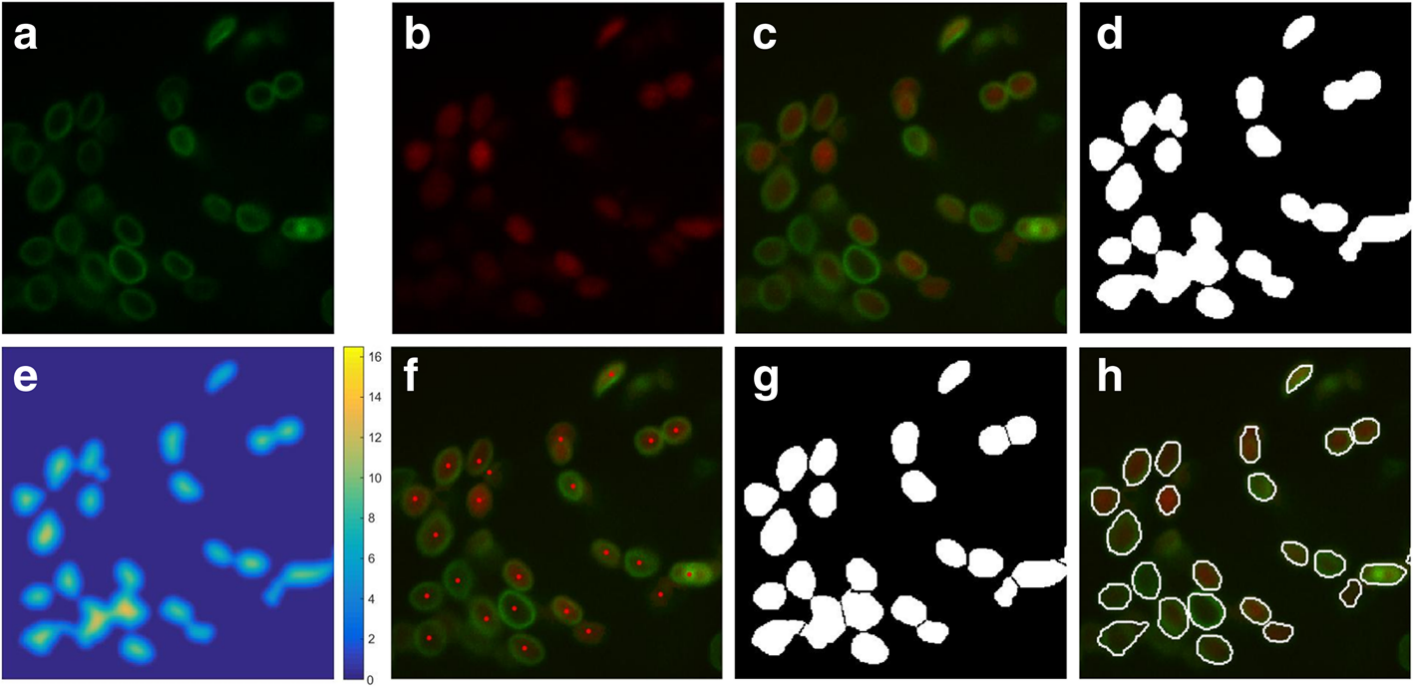
The process of nuclear segmentation methods. **a** The raw green-channel image. **b** The raw red-channel image. **c** The fused image of (**a**) and (**b**). **d** The binary image after thresholding. **e** The distance map of (**d**) (lighter color indicates higher value). **f** The fused image with seeds. **g** The binary image after seed-based cluster splitting (too small and dark nuclear regions are excluded). **h** Final result of the nuclear segmentation with white nuclear boundaries

First we use Otsu’s method to calculate the global binarization threshold of the green-channel image (*I*_*g*_) and get the binary image (*I*_*b*_). *I*_*b*_ is the filter kernel for the red-channel image (*I*_*r*_). These two images are merged by:

$$I_{g} \times \frac{P \times W_{g}}{P_{g}} + I_{r} \cdot I_{b} \times \frac{P \times W_{r}}{P_{r}} $$

where *P* is the maximal intensity of all imaging data. *W*_*g*_ and *W*_*r*_ are the weights of the green-channel image and the red-channel image. We set *W*_*g*_ and *W*_*r*_ to 0.6 and 0.4, respectively. *P*_*g*_ and *P*_*r*_ are the maximal intensity of *I*_*g*_ and *I*_*r*_, respectively. An example of image fusion is shown in Fig. 4(a-c). After that, the intensities of nuclei in current focus plane are enhanced and those not in current plane are diminished. Thus, the nuclear boundaries are sharpened, allowing for more accurate segmentation.

#### Thresholding segmentation

Our image fusion makes the segmentation much easier so that a simple threshold method is efficient for binarization. We first roughly extract the nucleus from the fused image by using Otsu’s method to obtain a suitable threshold [21]. However, this method is not always effective because of the out-of-focal-plane noise during imaging. When Otsu’s method fails, local thresholding is applied to binarize images by computing a threshold at every center pixel of every 701×701 pixels region. The field of view (FOV) of the region is about 72×72*μ*m, the width of which is approximately the width of the worm body. Generally, most of the images can be properly binarized. Figure 4(d) shows a binary image example.

#### Seed-based segmentation

**Fig. 5:**
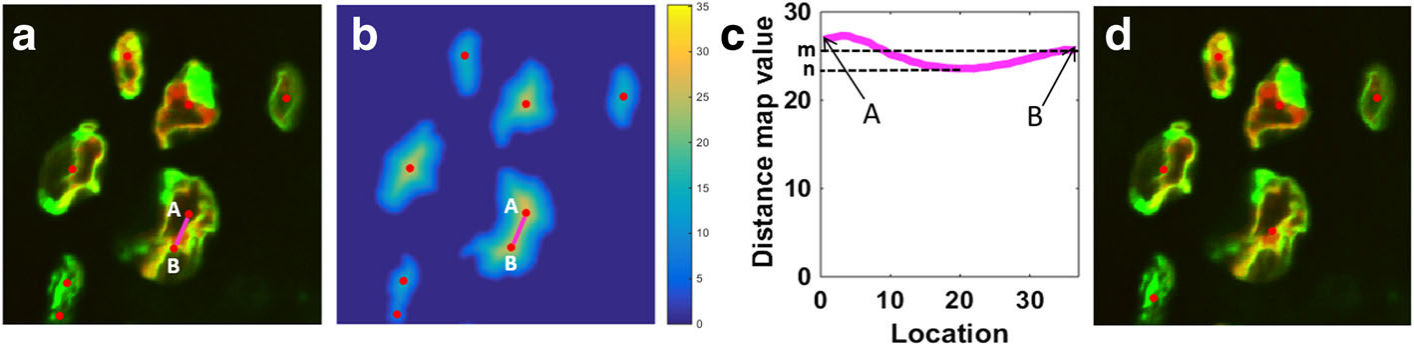
Seeds mergence process. **a** More than one seeds in the nuclei. The red points indicate the seeds. The pink line is a straight line linking seed A and B. **b** Distance map of binary image of (**a**) (the indicators are the same as (**a**)). **c** The distance map value on the line AB. The x-axis is the pixel location on AB. The y-axis is the pixel’s value in distance map. **d** The image after seed mergence

We first transform the binary image to a distance map *D*. The gray level of each pixel in *D* is the Euclidean distance between itself and the nearest zero pixel of binary image. Figure 4(e) shows an example of a distance map. Then we apply Gaussian smoothing to smooth small fluctuations in *D* and adopt the local maximums as seeds, which indicate the locations of the nuclei. But the problem is that long or irregular regions have more than one seed, like Fig. 5(a). So we need to merge these seeds.

To merge the seeds, we compare the lower value (*m*) of two seeds (A and B in Fig. 5) and the minimal value (*n*) on the line (the pink line in Fig. 5) between two seeds. If *n*>*m*×*r*, these two seeds would be merged into one seed located at their midpoint. *r* is a value close to the ratio of the lowest and highest nuclear intensity. It is set to 0.928 for our data set. Figures 4(f) and 5(d) shows the fused image that has only one seed in each nucleus after seeds mergence. The next step is to split the clustered region based on the seeds. We compute the distance transformation and force the value of the seed as negative infinity. And finally we compute the watershed transform of the modified distance map. Figure 4(g) gives the cluster splitting results.

#### Precise segmentation

**Fig. 6:**
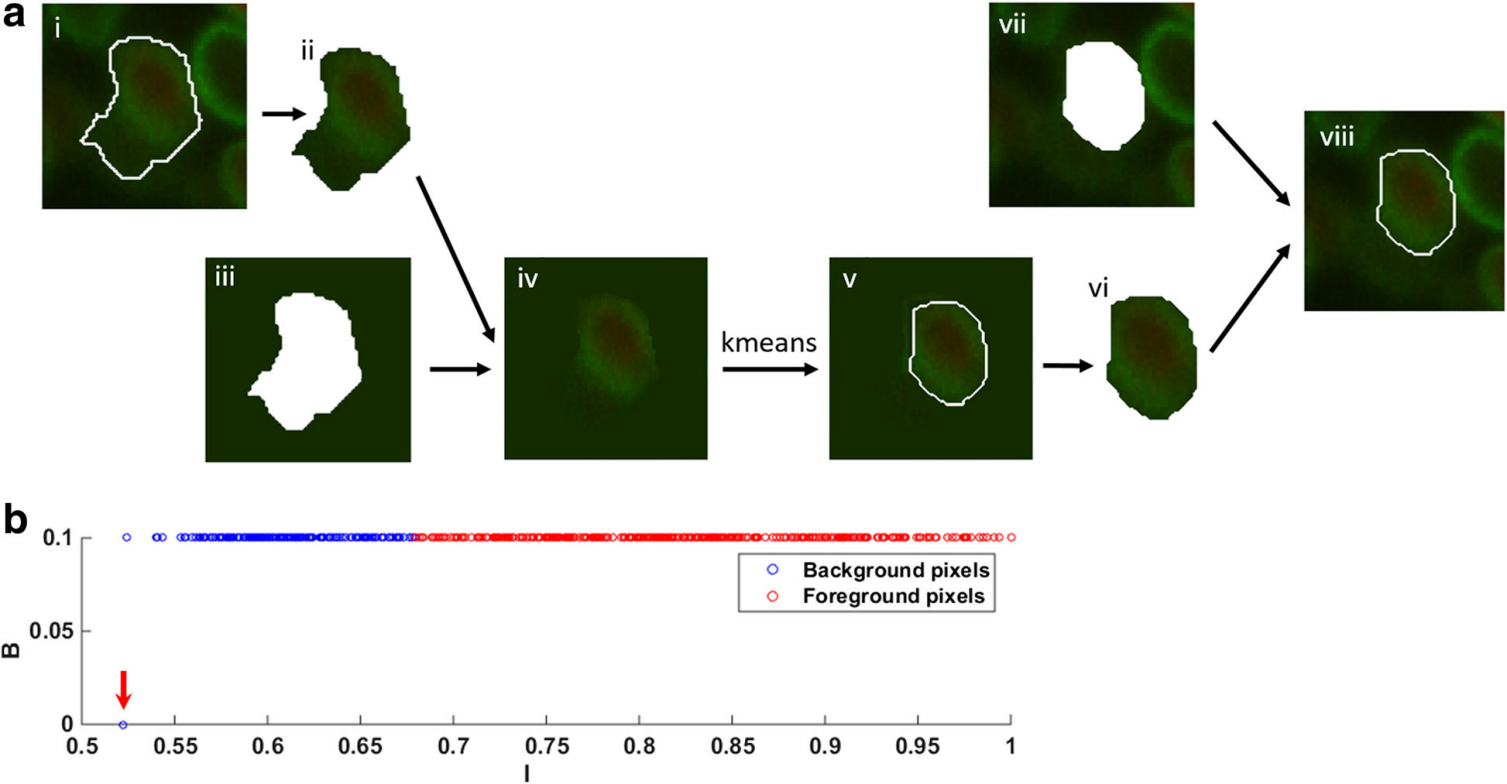
Precise segmentation process. **a** The precise segmentation pipeline. i is the roughly segmented nucleus on the fused image. ii is the nucleus extracted from i. iii is a pure intensity background we constructed, whose gray value is the mean intensity of the boundary (the white line in i). iv is the image combined by ii and iii. v shows the new nuclear boundary. vi is the extracted nucleus. vii is the original background in fused image. viii is the final result of precise segmentation. **b** The result of k-means clustering. The x-axis is *I* and the y-axis is *B*. The blue circles represent the background pixels and the red ones represent the foreground pixels. The blue circle that the red arrow points to denotes all the pixels in iii. These pixels have the same *I* and *B* values

In this step, the rough boundaries of nuclei are modified to be more precise. Based on the results of last step, we construct windows for each nucleus on the fused image. As shown in Fig. 6(a), we extract the roughly segmented nucleus (Fig. 6(a)-ii) from fused image and combine it with a pure intensity background (Fig. 6(a)-iii), where intensity of all pixels is the mean intensity of the pixels on rough boundary of the nucleus (the white line in Fig. 6(a)-i). Then the k-means algorithm [22] is applied to cluster the pixels in a two-dimensional space, *I* and *B*. *I* is the value of pixels in the newly constructed window (Fig. 6(a)-iv) multiplied by weight *w*_1_, which is the reciprocal of maximum value in the window. And *B* is the value of pixels in binary image multiplied by weight *w*_2_, which is 0.4 in our experiment. Figure 6(b) shows that all the pixels are clustered into two groups. The red and blue circles correspond to the background and foreground pixels. After all of the nuclei are processed as above, the precise segmentation is completed. Figure 4(h) shows the final segmentation result.

### Classification

#### Feature extraction

**Fig. 7:**
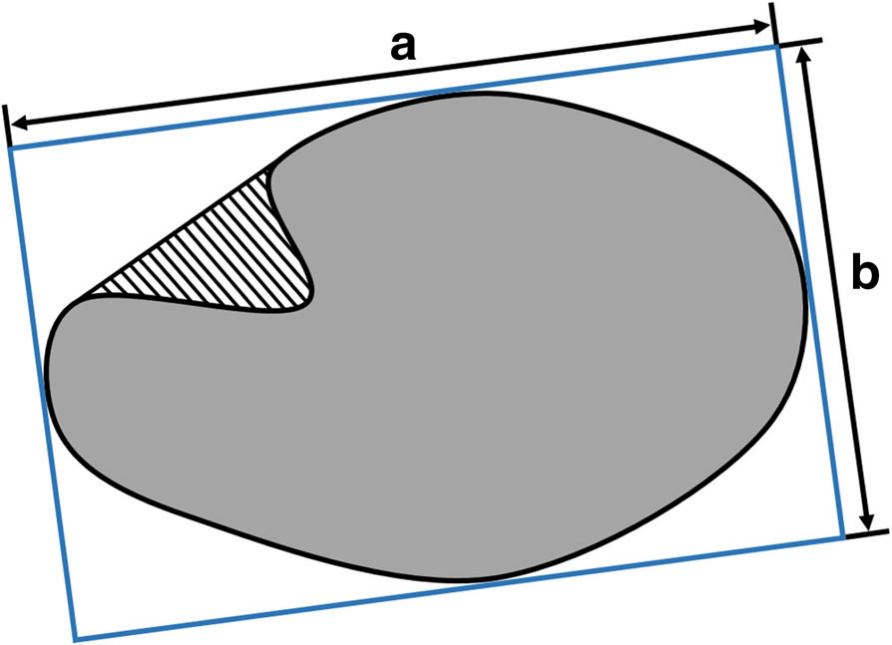
The convex hull and minimum enclosing rectangle of a nucleus. The pure gray region is a nucleus. The convex hull is the nucleus added to the region with stripped lines. The blue rectangle is the minimum enclosing rectangle of the nucleus, with length *a* and width *b*

After nuclear segmentation, we construct a feature set for classification. In this work, we extract geometric, intensity and texture features to describe the properties of nuclei. Geometric features are quantitative interpretations of nuclear shapes. Figure 7 shows some of the geometric features graphically. Intensity features are derived from the intensity histogram of each nucleus. Texture features are extracted from the gray level co-occurrence matrix (GLCM), a statistical measurement calculating how often pairs of pixel with specific values and in a specified spatial relationship occur in the nucleus [23]. We calculate GLCM of nuclei at directions of 0°, 45°, 90°, 135°. The offset of GLCM is 7, because the mean texture scale of nuclei in our data set is 7. To describe the GLCM features’ definition properly, we define *i* and *j* as the row and column of the co-occurrence matrix *C*, *p*(*i*,*j*) as the value in *C* of row *i* and column *j*. *μ*_*i*_, *μ*_*j*_ and *σ*_*i*_, *σ*_*j*_ denote the means and standard deviations of the row and column sums of *C*, respectively. The details are illustrated in Table 2. All of these features are extracted from both green-channel and red-channel images.

**Table 2:** Descriptions of geometric, intensity and texture features

Type	Feature	Description
Geometric features	area *A*	The number of pixels on the contour as well as the pixels enclosed by the contour.
	perimeter *P*	The number of pixels on the nuclear contour.
	circularity *C*	*C*=4*π* *A*/*P* ^2^, indicating the roundness of the nucleus.
	ellipticity	1−*b*/*a* (*a* and *b* are the length and width of minimum enclosing rectangle, shown in Fig. 7), measuring how much the nucleus deviates from being circular.
	solidity	*A*/*A*_*c*_ (*A*_*c*_ is the nuclear convex area measured by counting the number of pixels in the convex hull, as shown in Fig. 7).
	maximum curvature	The maximum of curvatures (The curvature at each boundary point is calculated by fitting a circle to that boundary point and the two points 10 boundary points away from it.).
	minimum curvature	The minimum of curvatures.
	std of curvature	The standard deviation of curvatures.
	mean curvature	The average absolute value of curvatures.
Intensity features	mean $\bar x$	Mean intensity of all pixels in the nuclei.
	variant *σ* ^2^	Variant of all pixels’ intensity in the nuclei.
	skewness	$\frac {1}{N-1}\sum _{i=1}^{N}\left (\frac {x_{i}-\bar x}{\sigma }\right)^{3}$ (*N* is the number of pixels in the nucleus). The negative or positive skewness means that most of the pixel values are concentrated at the right or left side of the histogram, respectively.
	kurtosis	$\frac {1}{N-1}\sum _{i=1}^{N}\left (\frac {x_{i}-\bar x}{\sigma }\right)^{4}$, describing whether the distribution is platykurtic or leptokurtic.
Texture features	contrast of GLCM	$\sum _{i,j}\left |i-j\right |^{2}p\left (i,j\right)$, measuring the intensity contrast between a pixel and its neighbor over the whole nucleus.
	correlation of GLCM	$\sum _{i,j}\frac {(i-\mu _{i})(j-\mu _{j})p(i,j)}{\sigma _{i}\sigma _{j}}$, measuring the dependencies between the nucleus image pixels.
	energy of GLCM	$\sum _{i,j} p(i,j)^{2}$, measuring the orderliness of texture. When the image is proficient orderly, energy value is high.
	homogeneity of GLCM	$\sum _{i,j}\frac {p(i,j)}{1+\left |i-j\right |}$, measuring the closeness of the distribution of elements in GLCM to its diagonal.

#### Feature selection

We get a 51-dimensional feature set from the previous section. But not all features contribute equally to the final nucleus classification. The redundant mutual relationships also generate incorrect classification results. In order to improve the performance of the classifiers and better understand the data, we need to reduce the feature dimension and find the significant features.

Since the range of feature values varies, some machine learning algorithms would not work properly without feature scaling and normalization. To ensure each feature contributes proportionately to the final distance metric, we firstly normalize each feature by projecting the minimum and maximum onto the range 0 and 1.

For feature selection, we first employ the minimum Redundancy Maximum Relevance (mRMR) feature selection scheme [24] to sort these features according to two distinct criteria. The first is “maximum relevance”, which selects features that have the highest mutual information with respect to the corresponding target class. The other is “minimum redundancy”, which ensures that the selected features have the minimum mutual information with other features. Constrained by these two variants, features that are highly related to the class labels and have the maximal dissimilarity with other features are at the top of the rank.

Then, we construct many feature subsets according to the rank. Each subsets contains the top *n* features. We input these subsets into the classifiers to discriminate the nuclei into different classes. We want to find the feature subset that makes the classifiers perform well and contains the least amount of features. The classifiers are the same with those in the following classification section.

#### Classification

The image data set of segmented nucleus includes not only the expected nuclei (the nuclei of four target tissues as mentioned above), but also the unexpected nuclei (the nuclei of other tissues or those can not be identified manually). All these nuclei are measured by selected features. These features are used in machine learning frameworks to train the classification models. This classification section is to discriminates the expected nuclei into the accurate tissue classes. The accuracy of unexpected nuclei is neglected because they are not our interests or we do not know which tissue they belong to certainly. All the classifiers are developed using scikit-learn, a machine learning library in Python [25]. The classification parameters can be found in Additional file 1.

In this stage, several machine learning algorithms are adopted and compared, including Support Vector Machine (SVM), Random Forest (RF) [26], *k*-Nearest Neighbor (*k*NN), Decision Tree(DT) and Neural Net(NN) [27].

The training data set of the classifiers is considered imbalanced since it exhibits an unequal distribution among four types of nuclei. To guarantee the classification accuracy of both the minority and majority classes, we set the weight of each class to $\sqrt {N_{total} / N_{i}}$, where *N*_*total*_ is the total sample amount of the training set and *N*_*i*_ is the sample amount of class *i*.

The optimal parameters are found exhaustively in the large grid of candidate parameter values using cross-validation [28]. We use 3-fold cross-validation to estimate the performance of classifiers with each parameter combination. In each estimating trial, the data set are randomly split into three parts, two of them are the training set *Tr* and the other one is the testing set *Te*. *Tr* is used to train the classifier with this parameter set. *Te* is classified by the classifier and the prediction result is compared with the true value. The final result is a score that calculated by the mean dot product of class accuracy and their weights. After testing the whole parameter set, we adopt the parameters that achieve the highest score in the classifiers.

An SVM classifies the data by finding an optimal hyperplane that separates data points of one class from other classes. The best hyperplane for SVM is the one with the largest margin between the classes, where margin is the distance from the decision surface to the support vectors. Our SVM classifier employs a linear kernel function and an one-against-one approach [29] to deal with the four-class problem.

Random Forest is a classification method that constructs a multitude of decision trees at training time. The output is the mode of the individual trees. During decision trees construction, we use information gain to measure the quality of a split and finally construct 19 trees in this forest.

*k*-NN is a non-parametric method where the input consists of *k* closest training examples in the feature space and the object is assigned to the label that is most common among its *k* nearest neighbors. We set *k* to 10 in our *k*-NN classifier. We use Manhattan distance to measure the distance between samples and use *k*-dimensional tree to compute the nearest neighbors [30].

Decision tree is a flow-chart-like structure, where each internal node denotes a test on an attribute, each branch represents the outcome of a test, and each leaf node holds a class label. Here we use Classification and Regression Trees (CART) algorithm to create decision tree. We utilize information gain to measure the quality of a split and choose the best random split.

For a neural network model, we use a multi-layer perceptron (MLP) which is a feed-forward artificial neural network and maps sets of input data onto a set of appropriate outputs. An MLP consists of multiple layers of nodes in a directed graph, where each layer fully connect to the next one. Except for the input nodes, each node is a neuron with a nonlinear activation function. It utilizes a supervised learning technique called back-propagation to train the network [31]. In our network, we have one input layer, one output layer and one hidden layer with 15 neurons. We apply Cross-Entropy as the loss function, *tanh* as the hidden layer activation function, and Softmax as the output function. For weight optimization, we use Adam, where the exponential decay rate for the first and second moment vector estimation are 0.9 and 0.999, and the value for numerical stability is 10^−8^. Also, we adopt L2 regularization to reduce over-fitting, where the penalty parameter is set to 0.001 and the learning rate is constantly kept at 0.001.

These classifiers are used both in feature selection and classification. In feature selection, all the classified nuclei are included in the final results. However, in classification, we measure the probabilities of the possible outcomes [32] and exclude the nuclei that have low classification probabilities (<90*%*) in the final results. Because high classification accuracy is more important than sensitivity in our study.

### Quantitative analysis

Many nuclei changes morphology during normal aging process. The aim of biologists is to find the nuclear morphological changing pathway and the differences between the pathways of two *C. elegans* strains (wild type and *daf-2(e1370)*). To show the effectiveness of our image processing method, we process a set of two-channel *C. elegans* nucleus-labeled fluorescence images using our automatic image processing method and obtain the image set of segmented and classified nuclei. As hypodermal nuclei change the architecture obviously during aging, we focus on hypodermal nuclei and calculate their area and solidity to demonstrate the effectiveness. The results are presented in the following section.

## Results and discussion

### Nuclear segmentation

To evaluate the segmentation performance, some nuclei are segmented by biologists manually, which is denoted as *G*. The automatic segmented nuclei by our methods are denoted as *S*. We evaluate the performance by calculating true-positive area (*TP*), false-positive area (*FP*) and false-negative area (*FN*) as follow:

$$TP = A_{G} \cap A_{S} $$

$$FP = A_{S} - A_{G} \cap A_{S} $$

$$FN = A_{G} - A_{G} \cap A_{S} $$

*A*_*G*_ is the number of pixels lying within the manual delineations of the nuclei. *A*_*S*_ is the number of pixels in the auto segmented boundary. To evaluate segmentation outcomes, we use precision *P* and sensitivity *S*:

$$P = \frac{TP}{TP+FP} $$

$$S = \frac{TP}{TP+FN} $$

**Fig. 8:**
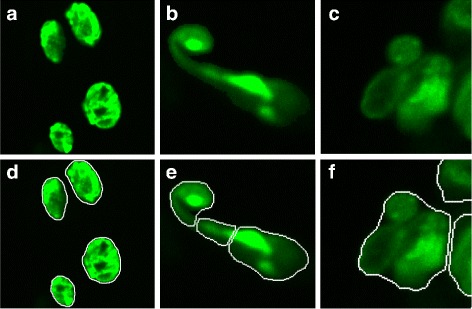
Three different segmentation cases. **a**-**c** The original green-channel images. **d** Correctly segmented nucleus. **e** Over-segmented nucleus. **f** Under-segmented nucleus

In order to show the importance of two-channel image fusion, we compare the segmentation results of using fused images and using only green-channel images. For nuclei of each different ages, we randomly select 60 nuclei, the amount of each tissue are proportional to the overall proportion of the whole nuclear data set (hypodermal : intestinal : muscle : neuronal ≈ 8 : 2 : 2 : 3). We calculate the average sensitivity and precision for segmented nuclei of different tissues and ages. The results are shown in Table 3. Comparing four tissues, performance on hypodermal nuclei is the best. Because hypodermal nuclei lie near the surface of *C. elegans* body, the intensity and contrast of hypodermal nuclei in images are higher. And they never cluster together. On the contrary, intestinal nuclei lie deeply in the worm body and neuronal nuclei usually cluster densely. Muscle and neuronal nuclei are smaller, thus they are more sensitive to small errors. Seeing the results of different ages, segmenting the old nuclei are slightly harder than young ones due to the distortion of old nuclei. In any case, the mean *P* and *S* of segmented nuclei using fused images are higher than using green-channel images. That is because red-channel images compensate the inside intensity of nuclei in green-channel images and enhance the contour contrast. Besides these evaluations, the following quantities are also measured and compared: total number of nuclei correctly segmented, over-segmented and under-segmented. After all the images are processed by our segmentation methods using green-channel images and two channel images, the segmented nuclei are manually classified into correctly/over/under segmented cases. Figure 8 shows an example of three segmentation cases. Table 4 shows the comparative segmentation results, including nuclear amount and percentage of each cases. 88.31% of the nuclei are correctly segmented by utilizing two-channel images, which is 6.24% higher than the single channel images. Consequently, the proposed segmentation method using two-channel image fusion gives a good partition of nuclei without losing the nuclear shape characteristics.

**Table 3:** Segmentation precision and sensitivity comparison between using one (green-channel) and two channel images

Tissue		Hypodermal		Intestinal		Muscle		Neuronal	
Channel		One	Two	One	Two	One	Two	One	Two
Day1	Precision	97.00%	99.19%	83.39%	99.64%	92.15%	99.02%	91.62%	98.11%
	Sensitivity	86.63%	91.76%	94.50%	91.89%	71.89%	81.80%	79.91%	81.44%
Day4	Precision	99.43%	99.59%	92.16%	99.38%	94.28%	98.25%	90.08%	97.42%
	Sensitivity	85.29%	89.16%	95.57%	92.83%	77.84%	85.88%	82.84%	84.94%
Day6	Precision	99.72%	97.86%	96.90%	97.43%	89.36%	96.13%	89.97%	98.37%
	Sensitivity	79.37%	92.48%	80.55%	95.63%	79.48%	90.33%	81.05%	83.46%
Day10	Precision	95.48%	98.83%	95.23%	96.54%	99.73%	99.39%	97.11%	98.51%
	Sensitivity	70.39%	95.30%	69.65%	94.23%	85.62%	87.45%	77.04%	92.16%
Day12	Precision	99.77%	98.59%	95.67%	95.22%	97.57%	98.02%	99.47%	98.99%
	Sensitivity	69.66%	92.79%	66.52%	93.38%	77.64%	90.40%	75.58%	87.83%
Day14	Precision	99.80%	99.21%	96.15%	95.64%	91.28%	92.99%	95.77%	96.91%
	Sensitivity	66.94%	93.86%	72.36%	92.22%	85.07%	89.46%	77.25%	84.00%
Day16	Precision	99.39%	99.44%	94.07%	94.34%	95.14%	95.65%	95.83%	96.81%
	Sensitivity	62.16%	91.81%	72.35%	91.23%	66.79%	77.96%	73.25%	81.97%
Sum	Precision	98.66%	98.96%	93.37%	96.88%	94.22%	97.06%	94.26%	97.87%
	Sensitivity	74.35%	92.45%	78.79%	93.06%	77.76%	86.18%	78.13%	85.11%

**Table 4:** Segmentation performance comparison between using one (green-channel) and two channel images

Type	Nuclei Amount	Correctly segmented	Over-segmented	Under-segmented
One Channel	10016	8220 (82.07%)	863 (8.62%)	933 (9.31%)
Two Channel	11154	9850 (88.31%)	330 (2.96%)	974 (8.73%)

### Classification

Using mRMR, features are sorted by the combination of the relevance to the target class and the relevance to other features. The top one in the rank has the highest relevance to target class and lowest relevance to other features. According to the rank, we construct 51 feature subsets. Each subset contains the top *n* features. The performance of classifiers using the feature subsets are evaluated by the mean class accuracy (MCA) of each classes, defined as $MCA = \frac {1}{n}\sum _{k=1}^{n}CA_{k}$, where *n* is the number of nuclear classes, *C* *A*_*k*_ is the classification accuracy of class *k*, calculated by *C*_*k*_/*N*_*k*_. *C*_*k*_ is the number of nuclei that are classified correctly as class *k*. *N*_*k*_ is the total nuclear number that are classified as class *k*.

**Fig. 9:**
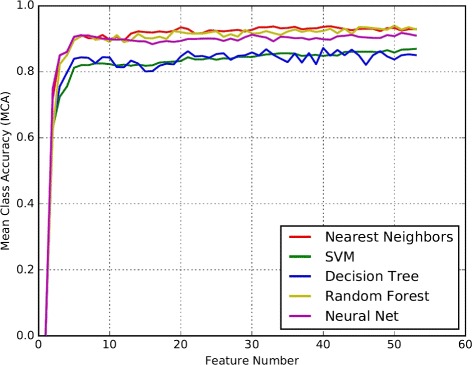
The performance of the classifiers with different subsets of features. The x axis, feature number, is the dimension of the feature subsets. The y axis is MCA. Five colors represent five classifiers

After sorting the features though mRMR, we use the classifiers to filter the features further. The performances of five classifiers with different subsets are shown in Fig. 9. According to the figure, the line zooms up from one feature to 5 features and levels off with slight oscillations until the end. It means that the most dominant factors for classification are the top 5 features. They are shape features (area, ellipticity, curvature mean and solidity) and texture feature (the homogeneity of GLCM at 90° on green-channel image). All these features agree with the empirical classification standards. The neuronal and muscle nuclei are usually smaller than the other two types. Neuronal nuclei are circle and muscle nuclei are elliptical. The intestinal nuclei typically have large area and high homogeneity. Hypodermal nuclei are quite complex. They have elliptical shape and smooth texture early, and have more irregular shapes and more variation in intensity distribution when they are old. Our shape and texture features can effectively distinguish four classes.

To compare the effectiveness of five classification algorithms, each classifier is evaluated by *MCA* and *C* *A*_*k*_. And the nuclei that have low classification probabilities (<90*%*) are excluded, because high classification accuracy is more important than sensitivity in our study. The classification results given by the five classifiers are listed in Table 5. In Table 5, it is clear that the Random Forest method performs better than other classifiers on our data set with the accuracy of 96.33%, 98.44%, 100.00%, 100.00% for hypodermal, muscle, neuronal and intestinal classes and 98.69% for MCA. Decision tree turns out to be the worst classifier among all, producing an MCA of 83.48% only. The reason why decision tree performs badly is that our features have high variance, making it difficult to find a clear and simple separation cut for the feature points. Beside decision tree, the other four classifiers produce perfect results in classifying muscle and neuron nuclei because these two types have obvious characteristics and scarcely change during the process of aging. The accuracy of hypodermal class is lower than others because they drastically change their shapes and textures when they are old.

**Table 5:** Accuracy of different types of nuclear classification and MCA of five classifiers (best classifier’s performances are written as bold text)

Method	Hypodermal	Muscle	Neuron	Intestine	MCA
SVM	93.77%	98.48%	100.00%	90.48%	95.68%
DT	87.27%	94.62%	85.14%	66.87%	83.48%
**RF**	**96.33%**	**98.44%**	**100.00%**	**100.00%**	**98.69%**
k-NN	96.33%	98.15%	100.00%	96.29%	97.69%
NN	94.74%	100.00%	100.00%	90.00%	96.19%

### Quantitative analysis

**Fig. 10:**
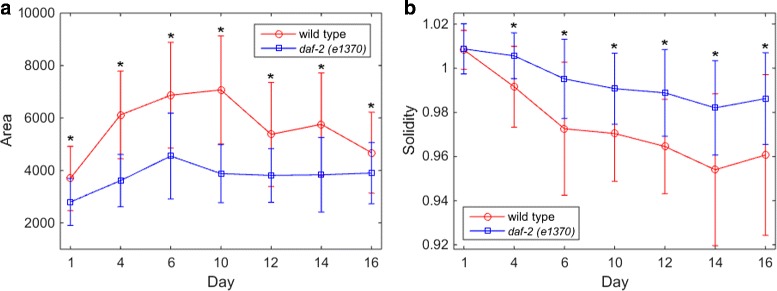
Quantification of age-dependent morphological changes for hypodermal nuclei in two strains. Area (**a**) and solidity (**b**) of wild type and *daf-2* hypodermal nuclei from adult day 1 to day 16. Data are the mean ± SD of all nuclei per time point. *P <0.0001, Welch’s t-test

The quantification results of age-dependent hypodermal nuclear morphological changes of two *C. elegans* strains are shown in Fig. 10. At 20°C, wild type worms have an average lifespan of about 20 days, and the *daf-2(e1370)* animals live twice as long the wild type [33]. From adult day 1 to day 16, the size of wild type hypodermal nuclei first increases and then decreases, forming a bell-shaped trend line. At its peak on adult day 10, the nuclear area is about twice as big as that on adult day 1. Over the same period, the change in the size of the *daf-2* hypodermal nuclei is far less than that of the wild type. And for animals of the same age, the *daf-2* nuclei are always smaller than those of the wild type (Fig. 10(a)). The *daf-2* hypodermal nuclei are also better at maintaining a smooth, round shape (measured as solidity) than their wild type counterparts, shown as a slow solidity decrease in the former and a faster and greater decrease in the latter (Fig. 10(b)). We propose that the smaller size and resistance to age-dependent changes are characteristics of the *daf-2* hypodermal nuclei, and they may be positively associated with *daf-2* longevity.

## Conclusions

In this paper, we proposed an integrated *C. elegans* nucleus-labeled fluorescence image processing method that consisted of nuclear segmentation, feature extraction, feature selection and classification.

Accurate nuclear segmentation method was achieved on fused two-channel images with seed-based cluster splitting and k-means. It overcame the difficulties of fuzzy nuclear boundaries and clustered nuclei segmentation and finally produced a high precision compared with manual segmentation result. Next, three groups of nuclear features were extracted to describe the nuclei, among which only five features were selected by mRMR for classifiers. Then, several popular classification techniques were compared using these features and the result indicated that Random Forest was the best classifier for our data set with an MCA of 98.69%. Finally, area and solidity of hypodermal nuclei were calculated and suggested that hypodermal nuclei in wild type were larger and more irregular than those in *daf-2(e1370)*. Smaller hypodermal nuclear area and smooth shape changes may be related to *daf-2* longevity.

The nuclei are three dimensional, and their locations and orientations vary in *C. elegans* body. Although some out-of-focus nuclei were filtered by fusion of two-channel images, we could not ensure that all the nuclei are in their optimal focus plane. However, the error caused by out-of-plane was most likely evenly distributed and unlikely to alter the conclusion that can be made from the comparison between time points or strains. In addition, the bright field images contained more information about the *C. elegans* strains and nuclei (for example, the size of the *C. elegans* body, the location of the nuclei and so on). In the future, we would like to capture and fuse more types of images for image processing and aging information mining.

*C. elegans* nucleus-labeled fluorescence images are very popular materials to study nuclear morphology during aging. Since there is no framework for this kind of images, we implement an automatic image processing method with segmentation, classification and quantification. Our method frees biologists from segmenting and classifying nuclei manually and subjectively. It reduces the quantification into a simple procedure. We have applied this method to the fluorescence image set and obtained some promising results, demonstrating its utilities in quantitative nuclear aging studies.

## Additional file

Additional file 1 Segmentation and classification of two-channel *C. elegans* nucleus-labeled fluorescence images. This file contains the following sections: S1-Limitations of the existing segmentation approaches; S2-Differences between green-channel and red-channel images; S3-Classification parameters. (PDF 354 kb)
