# Supplementary material for: Segmentation and classification of two-channel C. elegans nucleus-labeled fluorescence images
Source: BMC Bioinformatics. 2017 Sep 15;18:412. doi: 10.1186/s12859-017-1817-3 (PMC5602880; doi:10.1186/s12859-017-1817-3)
Supplement: Additional file 1 — Segmentation and classification of two-channel C. elegans nucleus-labeled fluorescence images. This file contains the following sections: S1-Limitations of the existing segmentation approaches; S2-Differences between green-channel and red-channel images; S3-Classification parameters. (PDF 354 kb) [file 12859_2017_1817_MOESM1_ESM.pdf]

Supplementary Text for: Segmentation and classification of two-channel *C. elegans* nucleus-labeled fluorescence images

Mengdi Zhao, Jie An, Haiwen Li, Jiazhi Zhang, Shang-Tong Li, Xue-Mei Li, Meng-Qiu Dong, Heng Mao*, Louis Tao*

S1 Limitations of the existing segmentation approaches

Watershed method

We find that watershed method causes over-segmentation, though we use some preprocessing methods, such as blurring and morphological operations. Our images contain both small and large nuclei, a single scale of preprocessing does not work effectively for all nuclei. Small operators cannot sufficiently smooth the large nuclei, while large operators over-smooth the small nuclei. Thus, we find watershed method over-segments some images that contain both small and large nuclei. Figure S1 shows the segmentation results of watershed and our method.

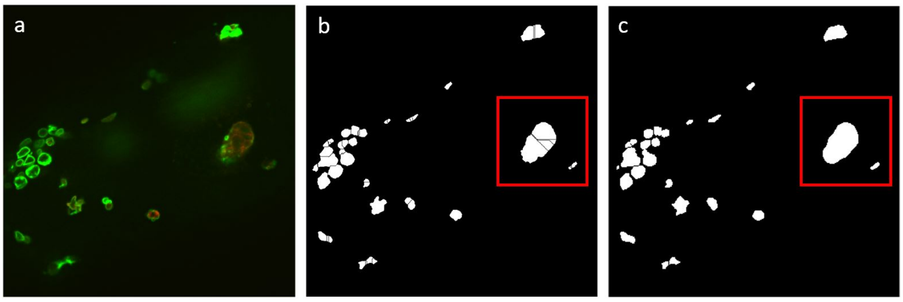

Figure S1: Comparative segmentation results between watershed and our methods. (a) The original image. (b) The segmentation result of watershed (the red box shows over-segmentation). (c) The segmentation result of our method.

Level set method

The level set method assumes that the gray level continuity or piecewise smoothness in the foreground. It is sensitive to the gradient ambiguities at the boundary and/or inside the nuclei. Our images contain nuclei of different ages, some of them have low intensity and complicated textures inside the nucleus, making the gradient disordered. Thus, this method is not suitable for our images.

Gradient vector flow tracking

The method in [1] is based on gradient vector flow tracking. It can segment densely packed, touching and not textured object; however, it cannot segment our textured nuclei properly. We find that the method often over-segments textured nuclei, and sometimes even results in holes in the nuclei, as shown in Figure S2 (b), especially the results in the red box. The primary reason is that the rough nuclear membrane texture in old worms produces disordered gradient flow magnitudes and directions, leading to the failure of gradient convergence.

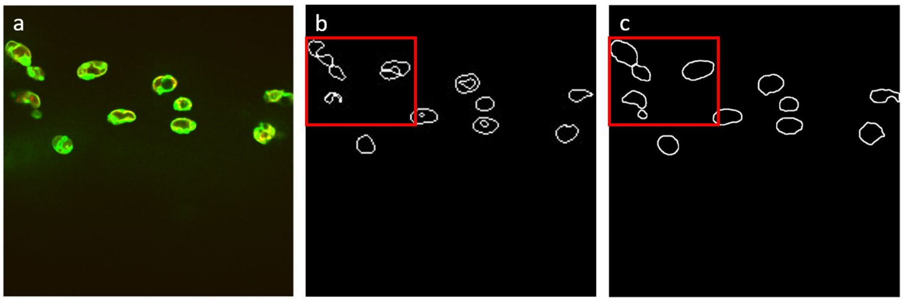

Figure S2: Comparative segmentation results between Li et al. and our method. (a) The original image. (b) The segmentation results of Li et al. (c) The segmentation results of our method.

Graph cut method

The graph cut method, which depends on the region connectivity of the foreground, cannot provide expected segmentation results because of the complicated nuclear textures. The method in [2] is based on the graph cut method. For comparison, we optimize the parameter described in this paper and find that for the images that contain both neuronal (small) and intestinal (large) nuclei, many nuclei are over-segmented, as shown in Figure S3.

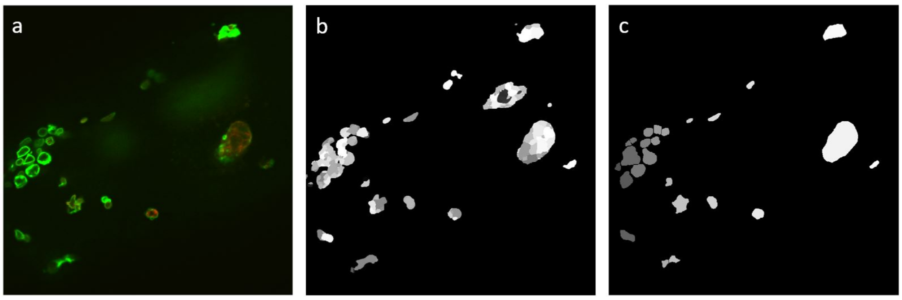

Figure S3: Comparative segmentation results between Al-Kofahi et al. and our method. (a) The original image. (b) The segmentation results of Al-Kofahi et al. (c) The segmentation results of our method.

S2 Differences between green-channel and red-channel images

The differences between green-channel and red-channel images are discussed below:

1. Green-channel images indicate the nuclear membrane, while red-channel images indicate the chromosome, which is inside the membrane.

2. Green-channel images are clear, while some red-channel images contain many bubble-like features, as shown in Figure S4 (a-b). These bubbles would affect the segmentation results, especially when they stick to the nuclei.

3. Green-channel images have higher signal-to-noise ratio (SNR) than red-channel images. Because the Green fluorescent protein that we use has much higher photoconversion efficiency. It is obvious that the highest intensity of Figure S4 (a) is higher than Figure S4 (b), thus, the former one has higher contrast.

4. Green-channel images of old worms do not contain strong fluorescent noise, while some red-channel images do. An example is shown in Figure S4 (c-d).

5. Green-channel images do not contain germ cell nuclei because transgenic *lmm-1::gfp* are silenced in germ cells. But some red-channel images contain these nuclei, which are not the interest nuclei for our aging studies. An example is shown in Figure S4 (e-f).

Therefore, green-channel images are more reliable than red channel images for segmentation.

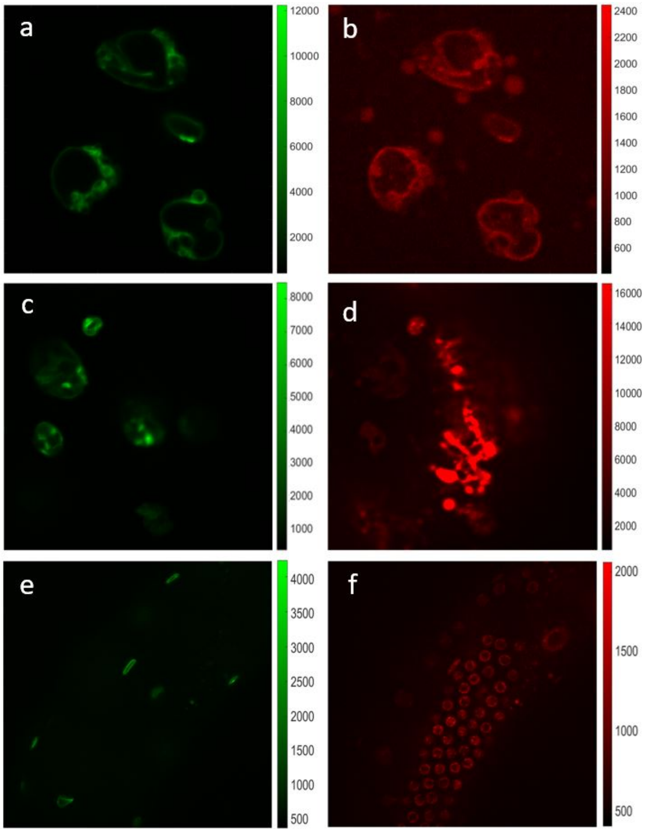

Figure S4: Comparison between green-channel and red-channel images. (a-b) The green-channel and red-channel image of example 1, (b) has lower SNR and contains some bubble-like features. (c-d) The green-channel and red-channel image of example 2 in Day 12, (d) has strong noise. (e-f) The green-channel and red-channel image of example 3 in Day 4, (f) contains many germ cell nuclei.

S3 Classification parameters

We constructed five classifiers using Support Vector Machine (SVM), Random Forest (RF), k-Nearest Neighbor (kNN), Decision Tree (DT) and Neural Net (NN). The classifiers were constructed using scikit-learn (http://scikit-learn.org/stable/), a machine learning library in Python. This section describes the details of the classifiers, including the range of parameter grid, the parameters and the functions we used for each classifier.

Range of parameter grid

We used GridSearchCV to find the optimal parameters, the parameter ranges of grid search were:

- SVM: ‘C’: [1:1:10] / ‘kernel’: linear, rbf / ‘decision_function’: ovo, ovr
- RF: ‘n_estimators’: [5:1:20] / ‘criterion’: gini, entropy / ‘max_features’: auto, sqrt, log2, None
- kNN: ‘n_neighbors’: [5:1:15] / ‘weights’: uniform, distance / ‘algorithm’: auto, ball_tree, kd_tree, brute / ‘p’: [1:1:3]
- DT: ‘criterion’: gini, entropy / ‘splitter’: best, random / ‘max_features’: auto, sqrt, log2, None
- NN: ‘hidden_layer_size’: [5:1:25] / ‘activation’: identity, logistic, tanh, relu / ‘solver’: lbfgs, sgd, adam / ‘learning_rate’: constant, invscaling, adaptive

Parameters and functions

- SVM: SVC (C=1, kernel = ‘linear’, decision_function_shape = ‘ovo’, probability = 1, class_weight = w)
- RF: RandomForestClassifier (n_estimators = 19, criterion = ‘entropy’, max_features = ‘auto’, class_weight = w)

- kNN: KNeighborsClassifier (n_neighbors = 10, weights =‘uniform’, algorithm = ‘auto’, p = 1)
- DT: DecisionTreeClassifier (criterion = ‘entropy’, max_features = None, splitter = ‘random’, class_weight = w)
- NN: MLPClassifier (hidden_layer_sizes = 15, activation = ‘tanh’, solver = ‘adam’, learning_rate = ‘constant’)

References

[1] Li G, Liu T, Tarokh A, Nie J, Guo L, Mara A, et al. 3D cell nuclei segmentation based on gradient flow tracking. BMC Cell Biol. 2007;8(1):40.

[2] Al-Kofahi Y, Lassoued W, Lee W, Roysam B. Improved automatic detection and segmentation of cell nuclei in histopathology images. IEEE T Bio-Med Eng. 2010;57(4):841-852.
